# A new technique for the analysis of metabolic pathways of cytidine analogues and cytidine deaminase activities in cells

**DOI:** 10.1038/s41598-023-47792-4

**Published:** 2023-11-22

**Authors:** Anna Ligasová, Barbora Piskláková, David Friedecký, Karel Koberna

**Affiliations:** 1 Institute of Molecular and Translational Medicine, Faculty of Medicine and Dentistry, Palacký University Olomouc, Olomouc, Czech Republic; 2 Laboratory of Inherited Metabolic Disorders, Department of Clinical Chemistry, Palacký University and University Hospital Olomouc, Olomouc, Czech Republic

**Keywords:** Haematological cancer, Microscopy

## Abstract

Deoxycytidine analogues (dCas) are widely used for the treatment of malignant diseases. They are commonly inactivated by cytidine deaminase (CDD), or by deoxycytidine monophosphate deaminase (dCMP deaminase). Additional metabolic pathways, such as phosphorylation, can substantially contribute to their (in)activation. Here, a new technique for the analysis of these pathways in cells is described. It is based on the use of 5-ethynyl 2′-deoxycytidine (EdC) and its conversion to 5-ethynyl 2′-deoxyuridine (EdU). Its use was tested for the estimation of the role of CDD and dCMP deaminase in five cancer and four non-cancer cell lines. The technique provides the possibility to address the aggregated impact of cytidine transporters, CDD, dCMP deaminase, and deoxycytidine kinase on EdC metabolism. Using this technique, we developed a quick and cheap method for the identification of cell lines exhibiting a lack of CDD activity. The data showed that in contrast to the cancer cells, all the non-cancer cells used in the study exhibited low, if any, CDD content and their cytidine deaminase activity can be exclusively attributed to dCMP deaminase. The technique also confirmed the importance of deoxycytidine kinase for dCas metabolism and indicated that dCMP deaminase can be fundamental in dCas deamination as well as CDD. Moreover, the described technique provides the possibility to perform the simultaneous testing of cytotoxicity and DNA replication activity.

## Introduction

Nucleoside analogues are largely used as drugs in the chemotherapy of malignant and viral diseases. Their application and effectiveness are considerably influenced by a wide range of mechanisms involving transport, phosphorylation, and catabolism, as well as by the mutual competition with natural nucleosides. Typical examples of such modified nucleosides are the analogues of 2′-deoxycytidine (dCas), such as 2′,2′-difluoro-2′-deoxycytidine (gemcitabine, dFdC), 1-β-D-arabinofuranosylcytosine (cytarabine, ara-C), or 5-aza-2′-deoxycytidine (DAC, decitabine, 5-aza-dC).

The key prerequisite of the therapeutic action of these dCas is their incorporation into DNA. On the other hand, the mechanism of this action is different. Gemcitabine, once incorporated into DNA, leads to premature chain termination after insertion of another nucleotide triphosphate. This non-terminal position of gemcitabine, referred to as masked chain termination, inhibits its removal by DNA repair enzymes, ultimately leading to apoptosis^1,2^. Gemcitabine induces cell cycle arrest particularly in the S phase, with higher concentrations potentially causing apoptosis of those cells in the G1 and G2/M phases^3^. Similarly, cytarabine kills cells undergoing DNA synthesis in the S-phase of the cell cycle, whereby its chemotherapeutic action is primarily associated with DNA fragmentation and chain termination^4^. The mechanism of action of decitabine depends on the dose. At high doses, decitabine produces DNA adducts that result in DNA synthesis arrest and cytotoxicity. At low doses, it induces gene expression profile changes that favour differentiation, reduce proliferation, and/or increase apoptosis^5,6^.

Gemcitabine is currently used for the first-line treatment of pancreatic adenocarcinomas and also for the treatment of various solid tumours^1^. It is assumed that gemcitabine is rapidly inactivated through deamination by cytidine deaminase (CDD, EC 3.5.4.5), or in the monophosphate form by deoxycytidine monophosphate deaminase (dCMP deaminase, EC 3.5.4.12)^7–9^.

Cytarabine is presently used in the therapy of haematological malignancies and is usually administered intravenously or subcutaneously at doses of 100–3000 mg/m^2^ in several dosing schedules. Although cytarabine is a cytidine analogue, the “up” orientation of the 2′-hydroxy group means it resembles 2′-deoxycytidine^10^. Human equilibrative nucleoside transporter (hENT1) is crucial for cytarabine uptake into a cell^11^. Once inside the cell, cytarabine is activated by three phosphorylation steps into the triphosphate form, which upon incorporation into DNA/RNA results in the inhibition of DNA and RNA synthesis, thereby triggering cell death^12,13^. Deoxycytidine kinase (dCK) is the rate-limiting enzyme that catalyses the first phosphorylation step to form cytarabine monophosphate, which is further phosphorylated into the triphosphate form. Inactivating enzymes in the metabolic pathway of cytarabine include 5′-nucleotidases (such as NT5C2 and NT5C3) and deaminases (including CDD and dCMP deaminase catalysing the formation of 1-β-D-arabinofuranosyluracil (cytarabine-uracil; cytarabine-U) and cytarabine-U monophosphate, respectively)^14,15^. In this respect, Gandhi, et al. demonstrated the accumulation of cytarabine-UTP in the circulating blast cells of six patients with acute myeloid leukaemia (AML) treated with cytarabine^16^. The intracellular concentrations ranged from 6 to 50 µM corresponding, to ~ 2.4–9.3% of the cytarabine-CTP peak concentrations^16^.

**Figure 1:**
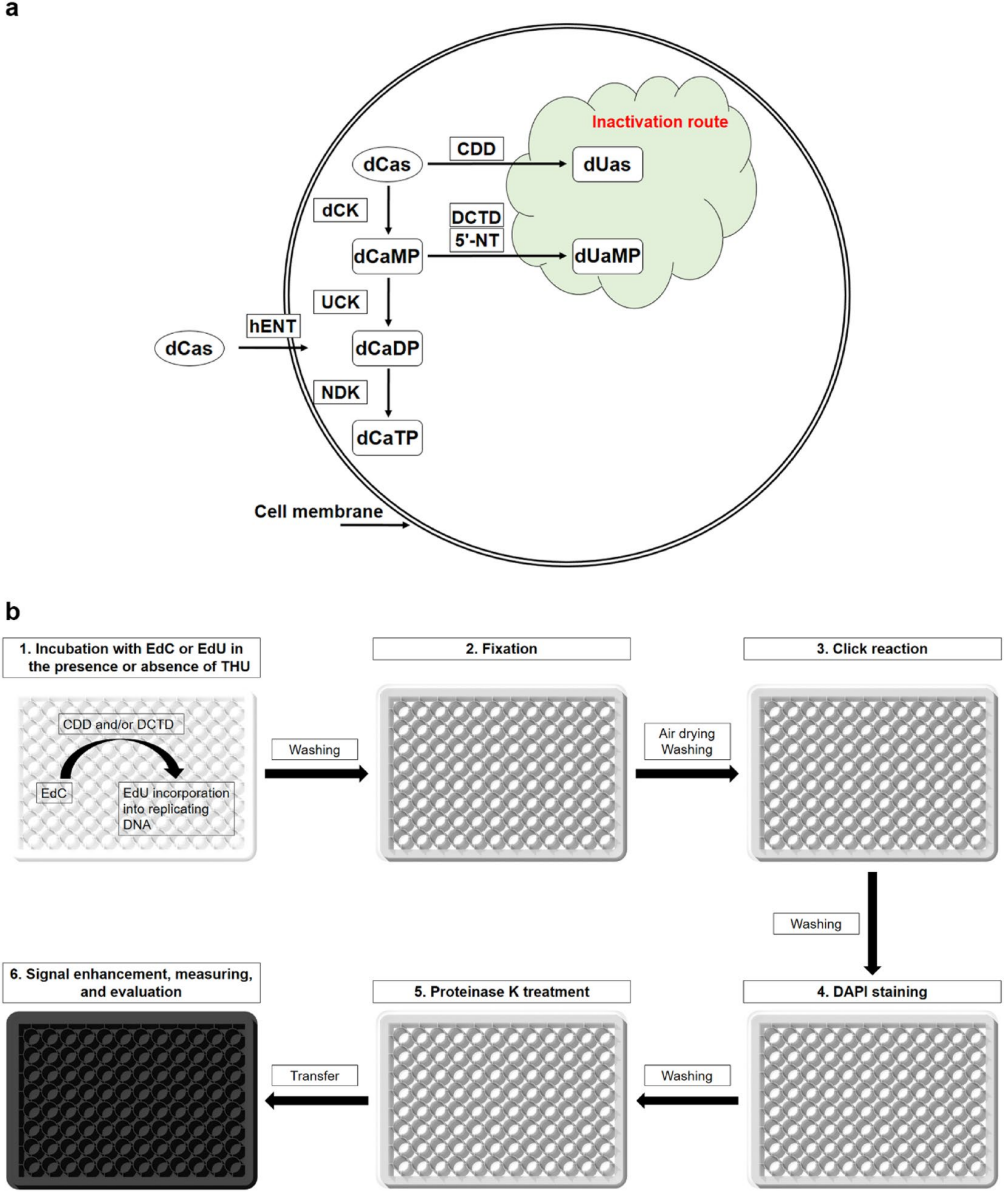
Schematic diagram of the basic dCas pathways (**a**) and the developed technique (**b**). (**a**) Simplified scheme of the activation and inactivation pathways of dCas. dUa—2′-deoxyuridine analogue; dCaMP, dCaDP, dCaTP—mono-, di-, triphosphate form of dCas; dUaMP—monophosphate form of dUa; dCK—deoxycytidine kinase; UCK—UMP-CMP kinase; NDK—nucleoside diphosphate kinase; CDD—cytidine deaminase; DCTD—dCMP deaminase; 5'-NT—5'-nucleotidases; and hENT—human equilibrative nucleoside transporter. (**b**) Schema of the developed assay for cytidine deaminase activity measurement by a plate reader. In the first step, EdC is deaminated and the product of deamination—EdU -incorporated into DNA. Fixation (second step) and air drying protect the release of the cellular DNA from the well bottoms during the following two steps and washing. Drying is of primary importance as it significantly increases protection from the mechanical and chemical treatments. During click reaction (third step), EdU is conjugated with the azide labelled with fluorochrome moiety. DAPI staining (fourth step) is used for DNA quantification. Proteinase K treatment (fifth step) results in the release of DNA into the solution. The DAPI signal is subsequently enhanced by SDS addition in the final step (sixth). In this step, cells are also transferred to the black well plate to maximise the fluorescent signals. The pipetting should be done carefully to avoid the formation of bubbles as they interfere with signal measurement. If the described technique is used for the simultaneous testing of cytotoxicity and replication activity, cells are incubated with EdU exclusively.

Decitabine is used in the therapy of myelodysplastic syndrome (MDS) and AML. It, like other dCas, is a target of metabolic pathways participating in the metabolism of 2′-deoxycytitidne, including deamination pathways^17^. A simplified scheme of the basic dCas pathways is shown in Fig. 1a.

As most of 2′-deoxyuridine analogues are relatively inactive when administrated as nucleosides, deamination has often been considered an inactivation route for dCas^10,18^. In this respect, some studies suggest that a combination therapy of gemcitabine and a specific inhibitor of CDD—tetrahydrouridine (THU)—is more effective for several types of cancer^19–22^. Deaminase inhibitors have also been used to prevent the inactivation of, and therefore increase the exposure to 5–fluoro-2′-deoxycytidine FdC;^23,24^ and cytarabine^25^.

From the published studies, it is evident that the results of the treatment using dCas reflect the interplay between many enzymatic reactions in blood plasma and cancer cells. This points to the importance of techniques that enable the quick assessment of dCas metabolism because such approaches can accelerate the research of the metabolism of the biologically active dCas, including those with potential pharmaceutical impact.

While the amount of proteins involved in dCas metabolism can be deduced from western blot analysis and the level of gene expression from RT-qPCR (reverse transcription followed by the quantitative polymerase chain reaction analysis) assay, CDD and dCMP deaminase activity is most frequently measured by approaches utilising mass spectroscopy. As all these approaches share the necessity to lyse cells and to analyse the activity in cell lysates^26,27^, these steps prolong the procedure and can, in the case of the analysis of enzyme activity, also result in unwanted effects during and after cell lysis. In this respect, an approach based on the visualisation of the product of the CDD and/or dCMP deaminase reaction directly in cells would make the analysis shorter and less prone to errors.

In this article, we present the results of a study focused on the possible utilisation of dCa with an ethynyl moiety, enabling its fast fluorescence detection in cells. Its use was tested for the estimation of the role of CDD and dCMP deaminase in different cell lines. The developed technique provides the possibility to analyse CDD and dCMP deaminase activity both before and after cell lysis and can facilitate the quick, cheap, and easy identification of cell lines missing CDD activity. Importantly, the developed technique can also be used for the simultaneous testing of cytotoxicity and DNA replication activity.

## Methods

### Cell lines and cell cultivation

Human cancer cells HeLa (cervix, adenocarcinoma; ATCC, CCL-2), 143B PML BK TK (143B TK; bone, osteosarcoma; ATCC, CRL-8304), 143B (bone, osteosarcoma; ATCC, CRL-830), NCI-H2009 (lung, adenocarcinoma), A549 (lung, carcinoma; both latter lines were a gift from Dr Marián Hajdúch, Institute of Molecular and Translational Medicine, Olomouc), HepG2 (liver, hepatocellular carcinoma), human immortalised diploid cells hTERT RPE-1 (hTERT-immortalised retinal pigment epithelial cells; both latter cell lines were a gift from Dr David Staněk, Institute of Molecular Genetics CAS, Prague), human diploid fibroblasts IMR-90 (lung, ATCC, CCL-186), MRC-5 cells (lung; a gift from Dr Marián Hajdúch, Institute of Molecular and Translational Medicine, Olomouc), and WI-38 (lung; a gift from Dr David Staněk, Institute of Molecular Genetics CAS, Prague) were used.

The HeLa, NCI-H2009, A549, and hTERT RPE-1 cells were cultured in Dulbecco’s modified Eagle’s medium (D-MEM) supplemented with 10% foetal bovine serum, 3.7 g/l sodium bicarbonate, and 50 µg/ml gentamicin. The HepG2, WI-38, and MRC-5 cells were cultured in Eagle’s minimum essential medium (E-MEM) supplemented with 10% foetal bovine serum, 1.5 g/l sodium bicarbonate, 1 mM sodium pyruvate, and 50 µg/ml gentamicin. The IMR-90 cells were cultured in E-MEM supplemented with 20% foetal bovine serum, 1.5 g/l sodium bicarbonate, 1 mM sodium pyruvate, and 50 µg/ml gentamicin. The 143B TK cells were cultured in D-MEM supplemented with 10% foetal bovine serum, 3.7 g/l sodium bicarbonate, HAT (0.1 mM hypoxanthine, 400 nM aminopterin, and 0.16 mM 2′-deoxythymidine; dT), and 50 µg/ml gentamicin. The 143B cells were cultured in D-MEM supplemented with 10% foetal bovine serum, 3.7 g/l sodium bicarbonate, 50 µg/ml gentamicin, and 0.015 mg/ml 5-bromo-2′-deoxyuridine (BrdU). The cells were cultured at 37 °C in a humidified atmosphere containing 5% CO_2_. All the cell lines were regularly tested for mycoplasma contamination by PCR and enzymatic detection^28^.

In the case of the 143B TK cells, the culture medium was exchanged for a HAT-free medium one week before the experiments. These cells have viral thymidine kinase (TK) and were established by the transfection of the parental 143B TK^−^ cells with the vector containing pML–1 plasmid, sequence from the BK virus, and hsv–1 TK gene. The transfected 143B PML BK TK cells stably express viral TK^29–31^.

### Incubation with EdU and EdC

Cells were seeded in 96-well plates, with exception to the border wells in rows A and H. The border wells were filled with the culture medium and served as blanks. Concurrently, cells were seeded in Petri dishes (10 cm in diameter) to prepare cell lysates. The following day, EdU and EdC were added to the culture medium. For the detailed description of the specific EdU/EdC concentrations, see Table 1.

**Table 1:** Detailed description of the EdU and EdC concentrations used.

	1	2	3	4	5	6	7	8	9	10	11	12
A	Culture medium											
B	Ctrl	0.4 µM EdU	2 µM EdU	10 µM EdU	50 µM EdU	250 µM EdU	0.4 µM EdU	2 µM EdU	10 µM EdU	50 µM EdU	250 µM EdU	Ctrl
C												
D												
E												
F												
G		10 µM EdC										
H	Culture medium											

The cells were then incubated at 37 °C in a humidified atmosphere containing 5% CO_2_ with or without the indicated concentrations of EdU or EdC for 4 h. If the CDD specific inhibitor—tetrahydrouridine (THU)—was used, the cells were incubated with 10 µM EdU or EdC alone or with 10 µM EdU or EdC and 50 µM THU.

### Samples treatment and EdU detection

After incubation, the cells were quickly rinsed with 1 × PBS buffer (two times) and fixed with 70% ethanol (200 µl per well) for 10 min at room temperature (RT). After fixation, the well plates were either sealed with a parafilm and stored in the freezer at − 20 °C, or the ethanol solution was removed and the samples air-dried (at least 30 min, RT). If the plates were stored in the freezer, the samples were tempered at RT for 30 min before ethanol removal and drying.

After drying, the samples were rinsed with deionised water (1 × 10 min, 450 rpm, 25 °C) and incubated in a solution of 10 mM NaCl, 10 mM hydrochinon, 2 mM CuSO_4_ and 10 µM 5-FAM azide (click solution, 30 min, RT). After the click reaction, the samples were properly washed with 20 mM Tris–HCl, pH 7 and 150 mM NaCl (Tris buffer; 4 × 10 min, 25 °C, 450 rpm). To minimise the impact of different numbers of cells per well, the samples were stained with DAPI (3 µM solution in Tris buffer) for 30 min at RT. The subsequent washing was performed first in a 20 mM citrate buffer, pH 5, 0.5 M NaCl, 0.2% Tween-20 (3 × 2 min, RT) and then with a Tris buffer (2 × 5 min, RT). After washing, the samples were checked using a fluorescence microscope. If less than 1,500 cells per well exhibited the EdU signal, the images were acquired and the signals for 5-FAM and DAPI evaluated. Otherwise, the cells were incubated in a solution of 0.1 mg/ml proteinase K in 50 mM Tris–HCl, pH 8 for 1 h at 37 °C. Finally, 80 µl of the solution was transferred into a black 96-well plate with 20 µl of 10% SDS in water and mixed by repeated pipetting. The signals for 5-FAM and DAPI were measured using a plate reader.

### Preparation of cell lysates and protein content determination

For the LC–MS analysis of deaminase activity and the amount of deaminases in various cell lines, cell lysates were prepared^32^. In brief, cells were plated onto a Petri dish (100 mm in diameter) and cultured until 70–80% confluence. After washing with 1 × PBS buffer and the ice-cold buffer A (10 mM Tris–HCl, pH 7.4, and 10 mM KCl), ice-cold buffer B (10 mM Tris–HCl, pH 7.4, 10 mM KCl, 0.5 mM EDTA, and protease inhibitors) was subsequently added and the cells incubated on ice for 20 min. The lysed cells were scraped from the dish surface, transferred to an ice-cold tempered Dounce homogeniser, and homogenised. The homogenate was centrifuged for 10 min at 12,000 × *g*, 4 °C. The liquid fraction was then transferred to a new, ice-cold tube and used for the measurements or divided into aliquots and stored at -80 °C^32^. The protein content was determined using the Pierce BCA protein assay kit (ThermoFisher Scientific) according to the manufacturer’s protocol^32^.

### SDS–PAGE, western blots

To evaluate the amount of CDD and dCMP deaminase in the cell lysates, SDS–PAGE and western blots were performed according to^29,32^. In brief, and if not stated otherwise, 10 µg of the total protein was resolved by SDS–PAGE at a constant voltage of 100 V for the first 10 min and 120 V for 1 h and 50 min. The proteins were then transferred to a nitrocellulose membrane (0.2 µm pore size, Bio-Rad) using a TE 22 Mighty Small Transfer Tank (Hoefer). The membrane was stained with Ponceau S, which served as a loading control^33^. After de-staining, the membrane was blocked in 5% BSA in TBS/T (Tris-buffered saline with 0.1% Tween-20) for 1 h and incubated with the primary antibody against CDD (D-5; Santa Cruz Biotechnologies, sc-365292) or dCMP deaminase (F-9; Santa Cruz Biotechnologies sc-376659) in 5% BSA and TBS/T overnight at 4 °C with agitation. The membranes were then washed with TBS/T and incubated with a peroxidase-labelled secondary antibody. The membranes were then washed and incubated briefly with Luminata Forte peroxidase substrate (Merck).

The Ponceau S signal was collected by the ChemiDoc MP Documentation system (Bio-Rad) and the chemiluminiscence signal by the Odyssey Fc Infrared imaging system (Li-Cor). The data were evaluated using ImageJ and Microsoft Excel software^29,32,34^.

### LC–MS analysis of CDD activity

The cell lysates were thawed on ice prior to their incubation with the selected substrates (cytidine, cytarabine, and EdC). To measure CDD activity, the lysates of nine cell lines were incubated in triplicate for each substrate. These lysates were diluted to a uniform concentration of total protein with the cold buffer (10 mM Tris–HCl, pH 7.4 and 10 mM KCl) and a substrate to a final volume of 70 µl. Due to the different specificity of CDD to the substrates used, these conditions were optimised to obtain a sufficiently strong product formation response (uridine, cytarabine-U, and EdU, respectively) while maintaining this enzymatic reaction in a burst phase. The final conditions are shown in Table 2. The reaction was started by adding the substrate. The mixture was then stirred, 30 µl of sample immediately taken and mixed with 120 µl of cold methanol (− 80 °C) to stop the reaction. The samples were shaken at 37 °C. After 10 or 30 min, 30 µl of sample was mixed with 120 µl of cold methanol. For sufficient protein precipitation, the samples were placed at − 80 °C overnight. The samples were subsequently centrifuged (12,000 × *g*, 0 °C) for 20 min and 100 µl of supernatant used for LC–MS analysis. A ten-point calibration of analytical standards was prepared by binary dilution series and analysed with the batch. The upper limit of quantification (ULOQ) for cytidine, uridine, cytarabine, and cytarabine-U was 2 µmol/l and for EdC and EdU, 4 µmol/l.

**Table 2:** Final incubation conditions of cell lysates with various substrates in a 70 µl mixture for measurement of CDD activity.

Protein concentration (µg/ml)	Substrate, concentration (µmol/l)	Incubation time (min)
50	Cytidine, 10	10
200	cytarabine, 10	10
400	EdC, 20	30

The analysis was performed on the HPLC instrument Exion LC (SCIEX, Framingham, MA, USA) using a Luna NH_2_ column (3 µm, 100 × 2 mm) (Phenomenex, Torrance, USA) and a mass spectrometer QTRAP 6500 + (SCIEX, Framingham, MA, USA) in scheduled MRM mode. The method parameters were used from the published works^29,35^, with only the MS parameters for cytarabine and cytarabine-U optimised using the analytical standards. The final selected reaction monitoring mass transitions were the following: *m/z* 242.8 > 110.0 for both cytarabine-U and uridine; 242.1 > 112.1 for both cytarabine and cytidine; 249.9 > 109.9 for EdC; 250.9 > 207.9 for EdU. The collision energy of each analyte was as follows: − 22 eV for cytarabine-U and cytidine, 19 eV for cytarabine and cytidine; − 20 eV for EdC; − 16 eV for EdU. The mass spectrometer settings were as follows: curtain gas, 40.0 psi; collision gas, medium; ion spray voltage, ± 4500 V; temperature, 400 °C; ion source gas 1 and 2, 40.0 psi.

### Data acquisition and data evaluation

An Infinite 200 Pro Plate Reader (Tecan) was used for the measurement of the fluorescence signal in the black 96-well plates. The fluorescence of 5-FAM was measured at 488 nm excitation wavelength and 520 nm emission wavelength. The fluorescence of DAPI was measured at 370 nm excitation wavelength and 460 nm emission wavelength.

Fluorescence images were acquired using an Olympus IX83 microscope (UPLFLN 2PH objective 10 × , NA 0.3) equipped with a Zyla camera (Andor) with a resolution of 1024 × 1024 pixels using acquisition software (CellSense Dimension, Olympus)^28,32^.

The data from the plate reader were analysed using Python 3.8^36^, NumPy^37^, Pandas^38^ libraries, and Microsoft Excel. The data from the fluorescence microscope were evaluated using ilastik 1.3.3post3^39^, CellProfiler 4.2.1^40,41^, Python 3.8^36^, NumPy^37^, Pandas^38^ libraries, and Microsoft Excel. EdU calibration curves and EdU concentrations derived from EdC were calculated using standard four parameter logistic non-linear regression, enabling the interpolation of the unknown values from the standard curve (GraphPad Prism 6). The data from western blots were analysed using Image J and Microsoft Excel^29,32,34^.

For LC–MS data acquisition and processing, Analyst® 1.7 and SCIEX OS 2.0 software (SCIEX, Framingham, MA, USA) were used. Substrate and product concentrations were calculated using a 10-point calibration curve. CDD activity was calculated from the increment of the product per unit time. In the blank samples, the physiological concentrations of cytidine and uridine were measured. The CDD activity was calculated according to the following equation (Eq. 1):

1 $$CDD activity= \frac{{c}_{e}-{c}_{0}}{t}$$

where *c*_*e*_ is the calculated concentration after 10 or 30 min incubation, *c*_0_ is the calculated concentration at time 0, and *t* is the incubation time.

The Pearson coefficients and p-values were calculated using the SciPy library^42^. The Levene test for homogeneity of variances was used for the comparison of variability of EdU/EdUMP production from EdC/EdCMP in the cancer and non-cancer cell lines. For the variance calculation within the cancer cells group and within the non-cancer cells group, Microsoft Excel was used. The Shapiro–Wilk test of normality and t-test were used in the statistical analysis. The final graphs were done in GraphPad Prism 6 and the final figures in Adobe Photoshop. If not stated otherwise, all the experiments were performed for three independent replicates. The data are presented as the mean values ± standard deviation (SD).

Figure 1a was drawn using Microsoft PowerPoint 2013, and Adobe Photoshop CS4 software, Fig. 1b using Rhinoceros 5, Microsoft PowerPoint 2013, and Adobe Photoshop CS4 software.

## Results

### Technique overview

#### Measurement by plate reader

Previously, it was shown that EdC is efficiently deaminated into EdU, phosphorylated, and incorporated into DNA^29^. Importantly, no or a very low amount of EdC was incorporated into DNA. As EdU can be easily visualised by fluorochrome azide after click reaction^43^, tests were conducted to see if the amount of EdU in DNA can serve as a monitoring system for EdC conversion.

The extent of incorporation was measured after click reaction with the azide dye. To reduce the impact of the different EdU phosphorylation and DNA incorporation rates among different cell lines, the EdU incorporation in cells incubated with the different concentrations of EdU was simultaneously measured. The production of EdU from EdC was estimated using non-linear regression, enabling the interpolation of the unknown values from the standard curve. A schematic diagram of the whole tested procedure is shown in Fig. 1b.

In the first step, cells were incubated with the various EdU concentrations (for calibration curve construction purposes) or 10 µM EdC (see Table 1). Simultaneously, tetrahydrouridine (THU) was added to some samples with 10 µM EdU or 10 µM EdC. In the control wells, cells were not incubated either with EdU or EdC. In the blank wells, cells were omitted. During this step, cells uptake EdU and EdC. Depending on the uptake, EdC phosphorylation and deaminase activity, EdC should be converted into EdU that is then incorporated into newly replicating DNA.

In the next step, samples were fixed with 70% ethanol. After fixation, the samples were either stored in the freezer at − 20 °C for up to several months or further processed and evaluated. If samples were stored in the freezer, they were tempered to RT for 30 min before further processing. After ethanol removal and sample drying, 30-min incubation in the click solution was performed to stain the incorporated EdU. The unbound azide dye, such as 5-FAM azide, was then washed out with a Tris buffer.

After washing, the cells were labelled with DAPI. DAPI staining enables cell quantification and therefore minimisation of the impact of the different numbers of cells in different wells^44^. In a further step, the samples were washed with a citrate buffer. This washing step was necessary for the removal of the unbound DAPI stain^44^. The samples were then washed in a Tris buffer and incubated in proteinase K solution. This incubation results in cell lysis. Finally, 80 µl of the solution was transferred into a black 96-well plate with 20 µl of 10% SDS in water and mixed by repeated pipetting. The signals for 5-FAM and DAPI were measured using a plate reader. The addition of SDS resulted in DAPI removal from the DNA and an increase in DAPI fluorescence by SDS micelle formation^44^. Although the measurement can be performed without SDS addition because DAPI fluorescence is enhanced by binding to DNA as well^44^, DAPI release from DNA results in insensitivity of the signal to DNA digestion through the eventual presence of nucleases, thereby providing the possibility to store samples at room temperature for at least several weeks.

**Figure 2:**
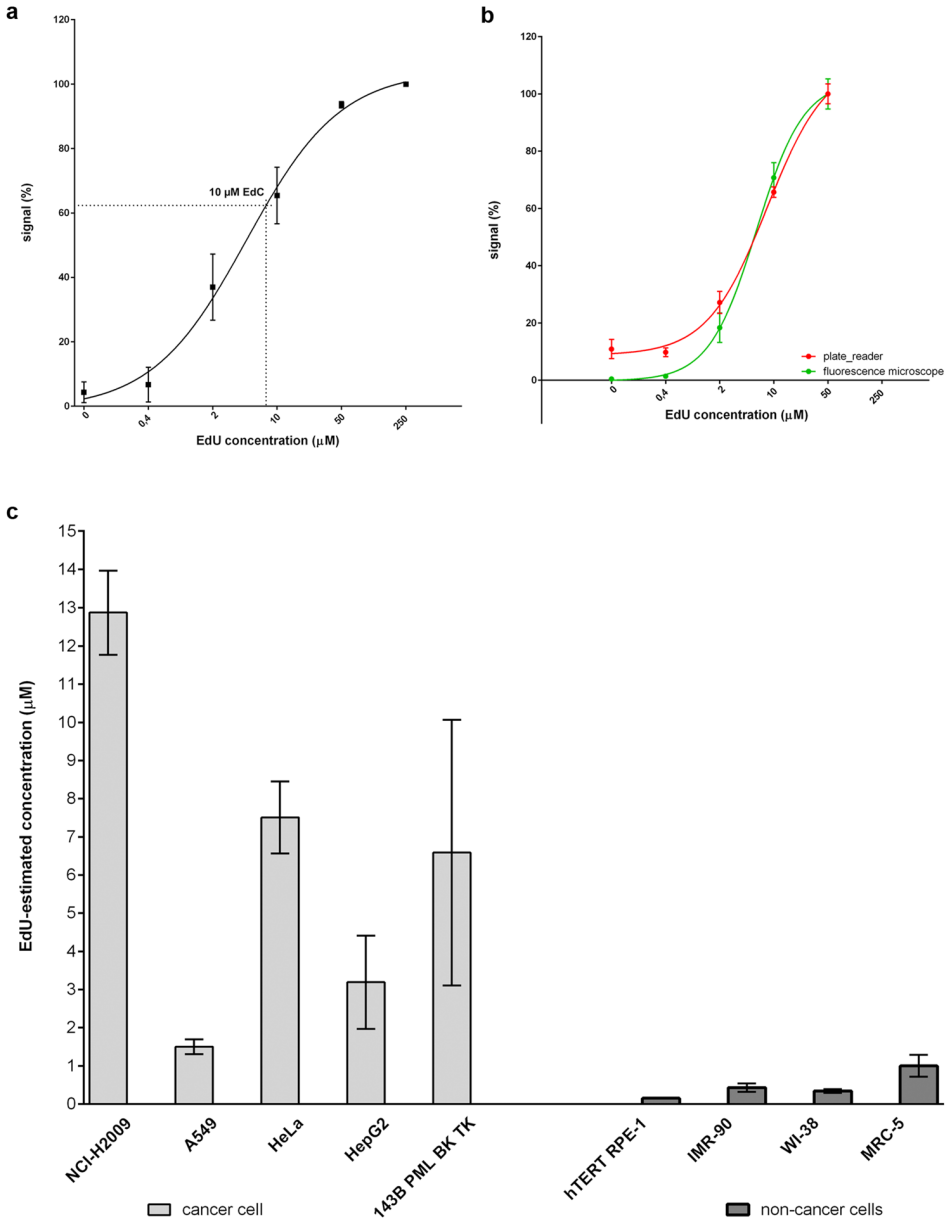
(**a**) An example of the EdU calibration curve and the interpolated EdU concentration of deaminated EdC as measured in HeLa cells. The data are presented as the mean values ± SD. (**b**) Comparison of EdU incorporation measurement by plate reader and fluorescence microscope. hTERT RPE-1 cells were labelled by various EdU concentrations and processed as described in the text. The data are presented as the mean values ± SD. (**c**) Comparison of overall CDD and dCMP deaminase activity based on EdC conversion in various cell lines. The values on the y axis correspond to the estimated concentration of EdU produced from 10 µM EdC. The data are presented as the mean values ± SD.

For evaluation purposes, the signal from the blank wells was averaged and subtracted from the signals measured in those wells with cells. Finally, the signal measured for EdU was divided by the DAPI signal to correct possible differences in the number of cells in specific samples. This value then served for the estimation of the production of EdU from 10 µM EdC and for the construction of the EdU calibration curve. If the production of EdU was estimated in THU non-treated cells, non-linear regression was used for the construction of the calibration curve. The production of EdU from EdC was calculated using GraphPad Prism 6 software interpolating the unknown values from the standard curve (standard curves to interpolate, sigmoidal, 4PL (four parameter logistic), *x* is log concentration). Figure 2a is an example of the non-linear regression for HeLa cells, including the interpolated value for EdC.

To observe the effect of THU on EdU production from EdC, the 10 µM EdC signal from THU-treated cells was first divided by the signal from samples incubated exclusively with 10 µM EdC. The corrected EdC + THU signal was then multiplied by the ratio of the signal from the cells treated exclusively with EdU and the cells treated with EdU and THU to minimise the impact of THU on EdU incorporation (Eq. 2).

2 $${THU}_{impact}(\%)= \frac{{EdC}_{THU}}{EdC}\times \frac{EdU}{{EdU}_{THU}}\times 100$$

The described technique can be quickly adapted for the simultaneous testing of cytotoxicity and DNA replication activity. In this case, cells in the first step are only incubated with EdU. For this purpose, 10 μM EdU is usually sufficient for replication signal detection.

#### Measurement by fluorescence microscope and comparison with plate reader

Although analysis of the signal by a plate reader is fast, if less than 1,500 replicating cells per well is analysed, it can result in a very low EdU signal and consequently in unreliable results. To solve this issue, the possibility of using a fluorescence microscope to evaluate the signal was tested. A very similar protocol was followed as for the plate reader, but with the following differences: immediately after labelling cells with DAPI and washing with a Tris buffer, the images were acquired using a fluorescence microscope. The DAPI signal was used to identify cell nuclei. The obtained images were evaluated using ilastik, CellProfiler, and Microsoft Excel. For the cytidine deaminase activity estimation, the ratio of the overall EdU nuclear signal and overall nuclear area was used. The calculated value was used for the estimation of EdU production from EdC and the obtained result compared to the simultaneously performed analysis using a plate reader (Fig. 2b). The data showed that both procedures provided similar data (Fig. 2b). In the case of hTERT RPE-1 cells, the calculated EdU production was 0.24 µM and 0.16 µM for the fluorescence microscope and plate reader, respectively. As both protocols share initial steps, the decision whether to use a plate reader or fluorescence microscope for the evaluation can be taken immediately prior to cell lysis. If necessary, both procedures can be performed using the same well plate.

From the results, it is evident that the procedure can be interrupted after fixation with ethanol and that samples can be stored in the freezer (− 20 °C) for several months. This is in agreement with the results of a previous study focused on the development of cytotoxicity assay based on a similar concept of DNA detection^44^. Alternatively, samples can be stored just before image acquisition. In this case, samples in the Tris buffer were stored overnight at 4 °C without significant effect on the signal measurement. If longer storage is necessary, the buffer should be exchanged for 20 mM Tris–HCl, pH 7.5, 50% glycerine in water and the samples stored in the freezer (− 20 °C).

Although the acquisition of the signal by fluorescence microscope does not require cell lysis by proteinase K and provides higher sensitivity, the necessity to acquire several images from every well, the application of an image segmentation algorithm, followed by image analysis and computation, substantially prolongs the procedure. From this point of view, the use of a plate reader is much faster because it provides the possibility to obtain data for final analysis within around 4 h after finishing the incubation step. The final analysis can be performed also very quickly because, for example, Python script or Microsoft Excel macro can be used for the signal evaluation and preparation of data for the non-linear regression analysis.

In the case of the use of a fluorescence microscope, the labelling procedure and acquisition can be performed in less than 4 h if an automatised microscopy station is used. However, the segmentation, image analysis and computation require additional time. If software such as ilastik, CellProfiler, and Python script are used, it requires more than 2 h to obtain the results from one 96-well plate. Importantly, as the analysis of THU-treated samples did not involve the analysis of samples with the serially diluted EdU, the image analysis was much quicker and enabled the results to be acquired in less than 3 h after incubation with EdC or EdU. In addition, we found that the washing step after the click reaction could be shortened to 20 min without effect on the background level.

It was also evident that independent of the method used for the analysis of the EdU signal, the described procedure can be stopped for a long time shortly after the incubation step (in ethanol) or just before measurement. Experiments can therefore be planned according to actual user needs.

### Variability of EdU/EdUMP production from EdC/EdCMP is higher in cancer than in non-cancer cell lines

The EdU production in various cell lines was compared. Cancer, diploid, and immortalised diploid cells were used for this purpose. The cancer cells were represented by HeLa, NCI-H2009, A549, 143B TK, and HepG2 cells, the diploid cells by IMR-90, MRC-5, and WI-38 cells, and the immortalised diploid cells by the hTERT RPE-1 cell line.

From the results obtained, it was clear that there is a very high variability in EdU/EdUMP production among particular cells (Fig. 2c, Table 3). This variability was much higher in the group of cancer cells (variance = 19.3) than in the group of non-cancer cells (variance = 0.099, *p* = 0.027, Levene test for homogeneity of variances). The 143B TK cells were excluded from the variability comparison and also from the next statistical evaluation because this cell line overexpresses the viral TK (hsv–1 TK gene, thymidine kinase) with the ability to phosphorylate deoxycytidine and its analogues^45^. The results also indicate that three cancer cell lines (NCI-H2009, HeLa, and HepG2 cells, mean = 7.85 ± 3.96) exhibited a much higher ability to produce EdU from EdC than the rest of the tested cell lines (mean = 0.68 ± 0.50, *p* = 0.013). This indicates that these three cell lines can exhibit high cytidine deaminase activity attributed to CDD and/or dCMP deaminase, although differences, for example, in EdC transport and kinase activity can also play an important role.

**Table 3:** Summary of the activity and the amount of CDD and dCMP deaminase (DCTD) in the tested cells.

Cell line	EdC conversion to EdU (µM ± SD)	Impact of THU on EdC conversion (% ± SD)	Amount of CDD (% ± SD)	Amount of DCTD (% ± SD)
NCI-H2009 †	12.87 ± 1.10	67.72 ± 4.35; *p* = 1.39e−06	75.25 ± 8.11	55.56 ± 8.13
A549 †	1.50 ± 0,20	69.42 ± 7.81; *p* = 6.69e−05	2. 46 ± 1.32	28.16 ± 6.44
HeLa †	7.51 ± 0.94	18.31 ± 1.87; *p* = 2.45e−08	42.29 ± 5.87	8.28 ± 5.42
HepG2 †	3.19 ± 1.22	16.26 ± 2.34; *p* = 4.61e−09	100	1.59 ± 1.36
143B TK	6.59 ± 3.48	103.00 ± 10.12; *p* = 4.00e−01	0.0 ± 0.00	67.64 ± 8.64
hTERT RPE-1	0.15 ± 0.003	106.40 ± 11.30; *p* = 4.60e−01	0.08 ± 0.08	80.20 ± 14.17
IMR-90	0.43 ± 0.11	99.16 ± 8.21; *p* = 6.00e−02	0.12 ± 0.20	52.85 ± 20.75
WI-38	0.34 ± 0.05	124.76 ± 17.23; *p* = 5.30e−01	0.00 ± 0.00	45.76 ± 23.63
MRC-5	1.00 ± 0.29	94.19 ± 23.03; *p* = 4.30e−01	0.00 ± 0.00	100

^†^*p* value ≤ 0.05 for comparative analysis of the impact of THU.

### Diploid cell lines are much less sensitive to treatment by THU than most cancer cell lines

THU is a selective inhibitor of CDD. The treatment of cells with THU should therefore result in a greater decrease in EdC conversion to EdU in cells with a high level of CDD activity than in cells with a high level of dCMP deaminase activity. In contrast to the estimation of the overall deaminase activity, this approach does not require calibration to minimise the effect of the different replication rates because the decrease is studied in the same cell line. For the estimation of the impact of THU, the ratio of the EdU signal from the cells incubated with EdC and THU and cells incubated exclusively with EdC was used. As the results also showed that THU can affect EdU incorporation into DNA, a correction for this effect was applied by multiplying the above mentioned ratio by the ratio of EdU signal from cells incubated with EdU and cells incubated with EdU and THU (Eq. 2).

**Figure 3:**
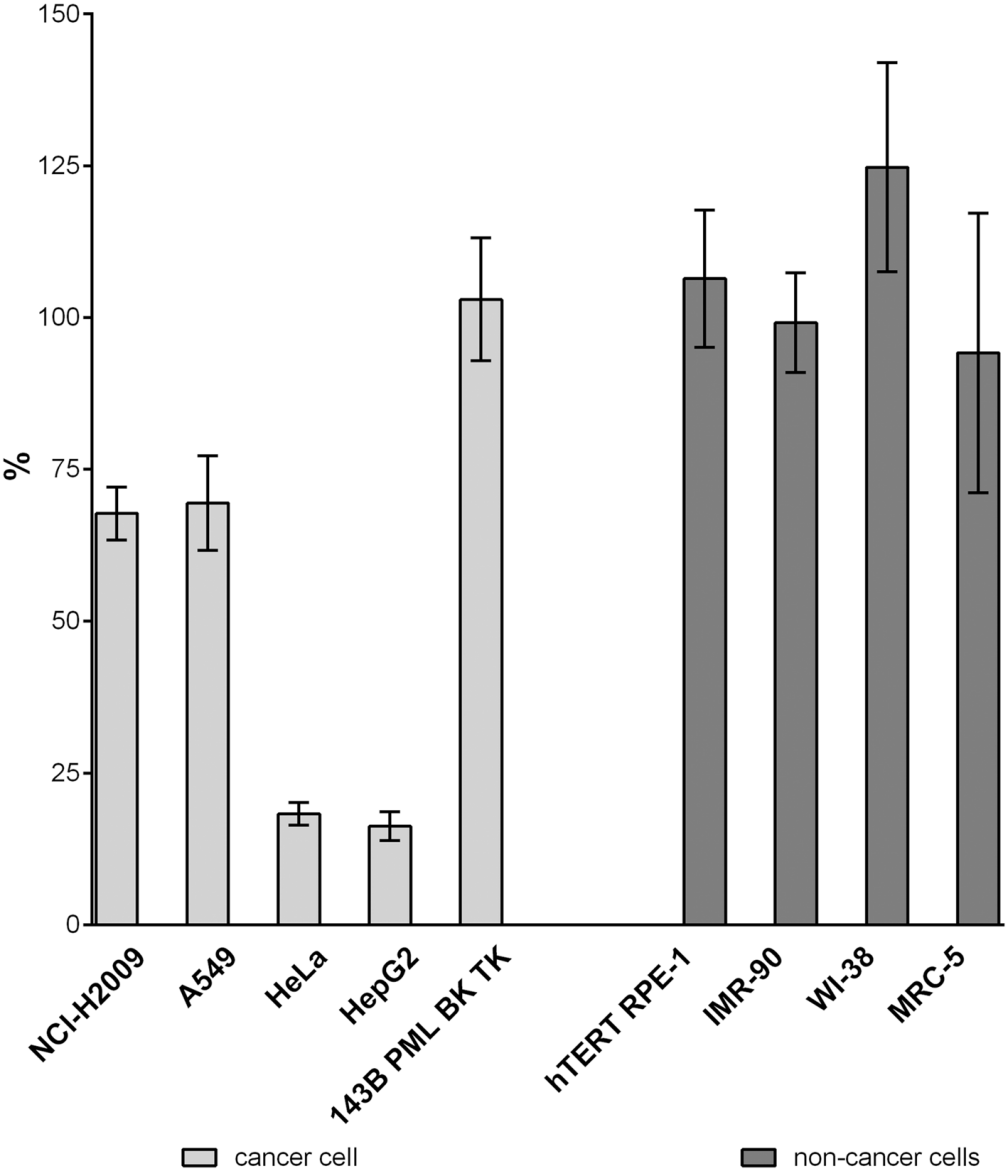
Impact of THU on EdC conversion in various cell lines. The data are related to the control (100%), untreated samples. The data are presented as the mean values ± SD.

The data showed that there is no significant effect of THU on the production of EdU from EdC in all the non-cancer cell lines and also in the 143B TK cell line (Fig. 3, Table 3). This indicates that these cell lines exhibit relatively high dCMP deaminase activity and very low, if any, CDD activity. Simultaneously, it proves that both EdC and EdCMP are effectively deaminated by cellular deaminases. These results also indicate that in HeLa and HepG2 cells, CDD is the main enzyme responsible for the conversion of EdC to EdU. The lower effect of THU on the NCI-H2009 and A549 cell lines indicates that both deaminases are present in these cell lines and that both play a role in EdUTP production.

### The developed method enables clear discrimination between cell lines with and without CDD activity

**Figure 4:**
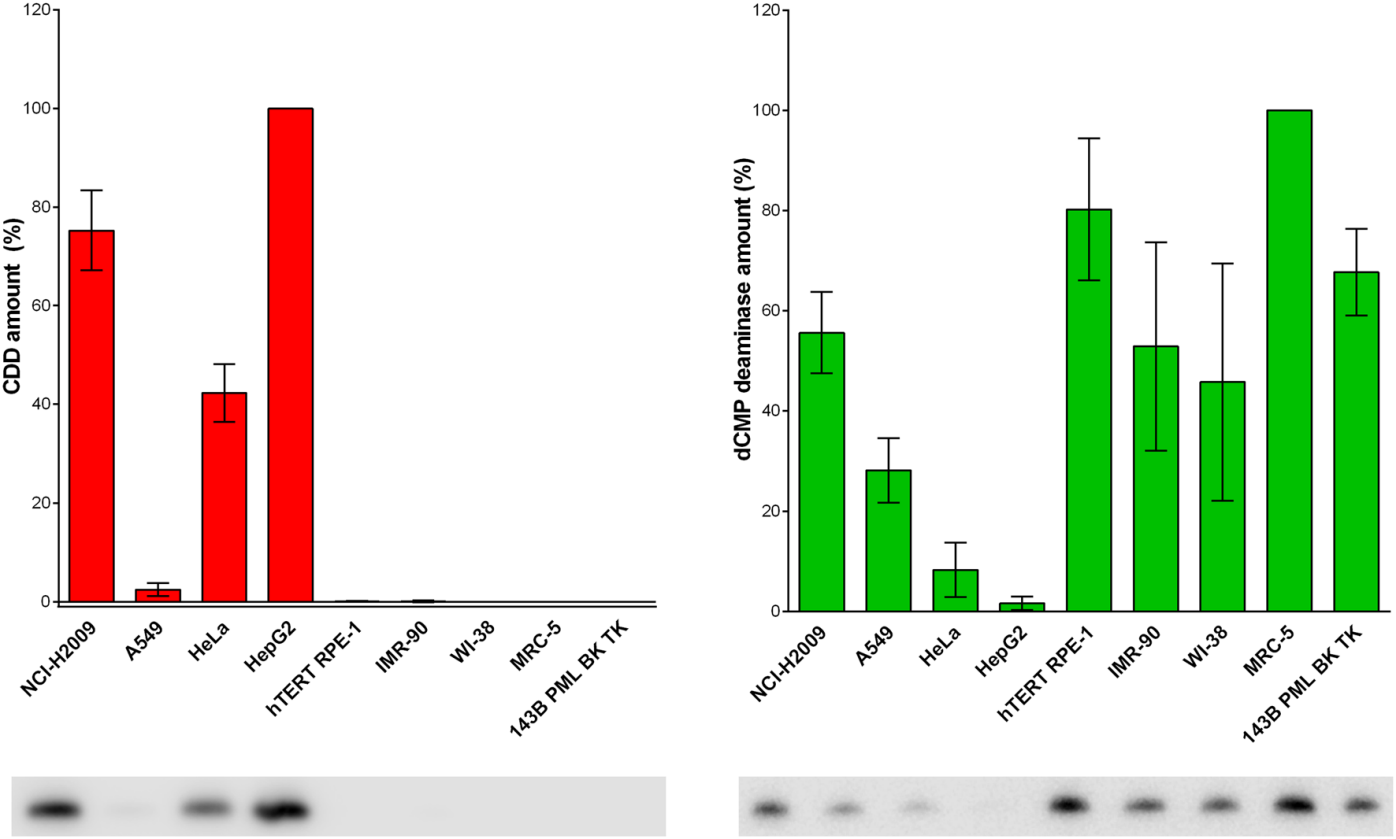
Comparison of the amount of CDD (**a**) and dCMP deaminase (**b**) in various cell lines. The data are normalised to the amount of CDD measured in HepG2 cells (**a**) and to the amount of dCMP deaminase in MRC-5 cells (**b**), both equal to 100%. The data are presented as the mean ± SD. Below both graphs are examples of the western blots corresponding to the specific cells displayed in the graph. In the supplementary data file, the original western blot images from the three independent experiments are shown with the marked western blots corresponding to the cropped images (Supplementary Fig. 1).

As the data indicated that some of the cell lines exhibit very low, if any, sensitivity to THU treatment, thereby denoting low or no amount of CDD, western blot analysis of CDD and dCMP deaminase content was performed. The data clearly revealed that the observed low sensitivity to THU treatment was accompanied by a lack of significant levels of CDD (Fig. 4a). Instead, these cell lines exhibited relatively high dCMP deaminase content (Fig. 4b). This is in complete agreement with the experiments conducted on the treatment of cells with THU. Unsurprisingly, the ratio of EdU incorporation without and with THU addition in cells incubated with EdC positively correlates with the amount of dCMP deaminase (Pearson correlation coefficient for all cell lines is R = 0.76, *p* = 0.016). This correlation was even stronger if only cancer cell lines were analysed (Pearson correlation coefficient R = 0.93, *p* = 0.02).

It was evident that cell lines containing CDD (NCI-H2009, HeLa, and HepG2) exhibited a higher rate of EdU incorporation than cell lines with dCMP deaminase only. The only exception was the 143B TK cell line (Figs. 2c and 4), where a much higher ability to produce EdU signal in DNA was observed than in the non-cancer cell lines, although 143B TK cells expressed dCMP deaminase similarly to the non-cancer cells. We hypothesised that this could be the result of the overexpression of viral TK. It is for this reason that EdU incorporation in the parental 143B cell line incubated with EdC was measured. This cell line lacks both the viral and natural cytoplasmic TK. As the lack of cytoplasmic TK disabled calibration of EdU incorporation due to the inefficient EdU phosphorylation to EdUMP, EdU signal after 4-h incubation with 10 µM EdC was compared. The 143B cells exhibited only 4.94% of the EdU signal derived from EdC compared to the signal measured in 143B TK cells. As the viral thymidine kinase can also phosphorylate deoxycytidine into its monophosphate form^45^, this probably results in a very high production of EdCMP, its deamination into EdUMP, and also to the very efficient incorporation of EdU into DNA during EdC incubation. These results strongly indicate that the dCMP deaminase in 143B TK cells can efficiently utilise the pool of EdCMP and convert it into EdUMP (see Fig. 3). From this point of view, our data show that dCMP deaminase can be as effective in the (in)activation route for dCas as CDD. In this respect, any dCas requiring incorporation into DNA for their biological effect have to be transformed into monophosphate form. This result also indicates that if dCas require transformation into the deaminated form for their biological effect, a higher effect can be expected in cells with CDD activity than in cells without it.

The data from western blot analysis clearly showed that the developed method based on EdC and THU provided a highly confidential system that enables the clear discrimination between cell lines with and without CDD activity. In this respect, all cell lines with no or a negligible decrease of EdU production after THU treatment, exhibited no CDD.

### CDD activity measured by LC–MS strongly correlates with CDD content

**Figure 5:**
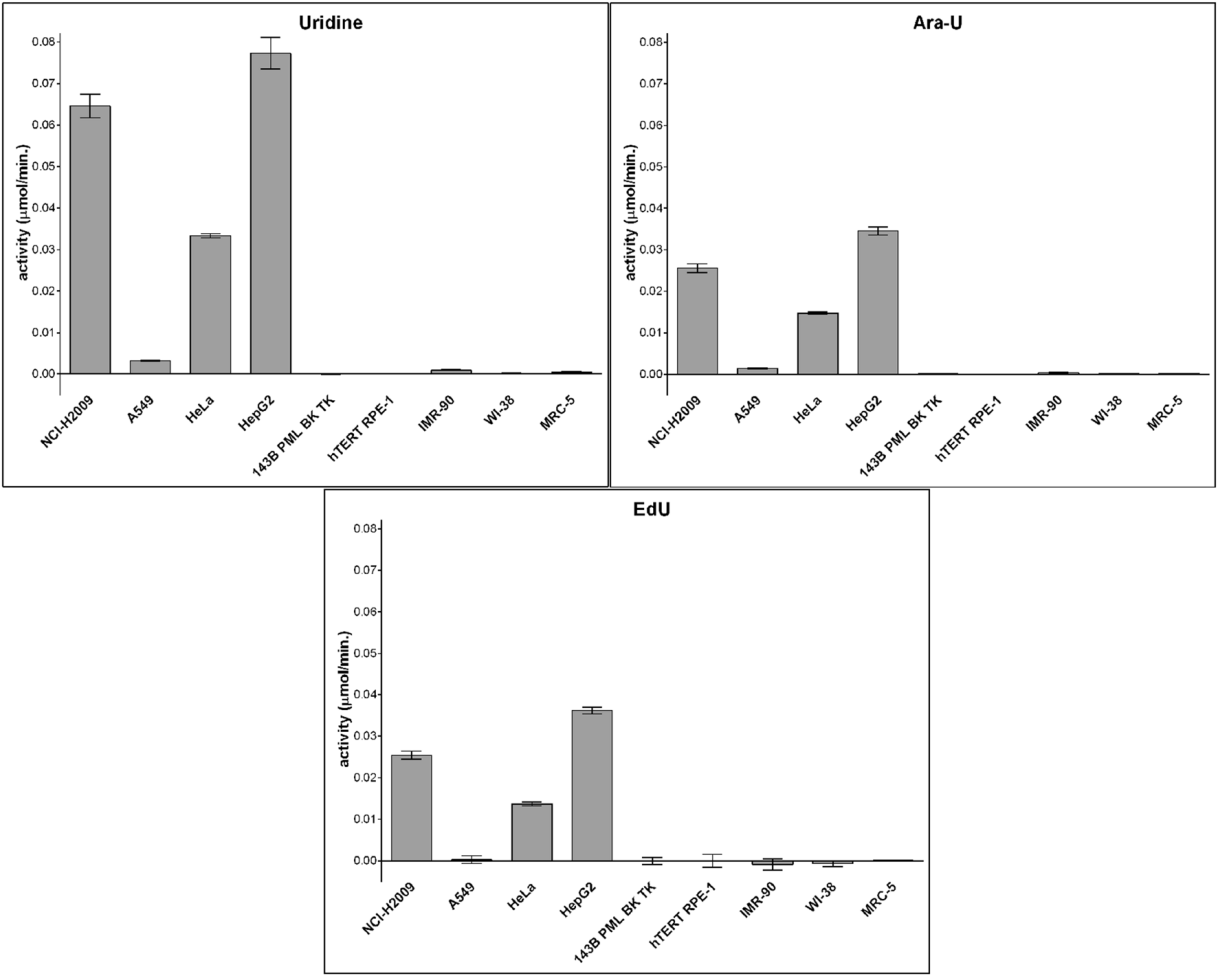
CDD activity measured using LC–MS. The data are presented as the mean values ± SD.

As this method does not allow separate measurement of CDD and dCMP deaminase activity and it was not clear how closely the content of CDD reflects CDD activity in the selected cell lines, the activity of CDD was measured in the same cell lysates using LC–MS (Fig. 5). Cell lysates (50 µg/ml, 200 µg/ml, or 400 µg/ml of the total protein for cytidine, cytarabine, and EdC, respectively) were incubated either with 10 µM cytidine or 10 µM cytarabine for 10 min, or with 20 µM EdC for 30 min. A higher EdC concentration and longer incubation time were used due to the lower sensitivity for EdU. In addition, the total protein concentration was adapted according to the reaction speed.

From the data obtained, it was clear that only cell lines containing a significant amount of CDD converted all three tested nucleosides to their uridine counterparts (Fig. 5, compare with Fig. 4). However, the substrate specificity was different. CDD exhibited the highest affinity to natural cytidine. 50 µg/ml of the total protein was enough to see the fast conversion of cytidine to uridine in 10 min. In the case of EdC, 400 µg/ml of the total protein and 30 min incubation was necessary to measure reliable results. The positive correlation between the activity and CDD content was extremely high (Pearson correlation coefficient R = 0.99, *p* < 0.001) for all three tested nucleosides. This supported the conclusion drawn concerning the role of CDD activities in different cell lines based on the analysis of EdU signal both in THU-treated and THU-non-treated cell lines. Although EdC and cytarabine deamination in cell lines exhibiting high levels of CDD was slower than the deamination of cytidine, such extremely high correlations indicate that there is no difference between the chosen cell lines and the effectiveness of the conversion by CDD. This was confirmed by cross correlation between the measured activities for cytidine, cytarabine, and EdC (Pearson correlation coefficient R = 0.99, *p* < 0.001).

## Discussion

dCas are commonly used for the treatment of malignant diseases. As they are also the target of various cellular enzymes, the effectiveness of these drugs depends on their activities. A new technique for the analysis of some pathways of dCas metabolism is described. The technique is based on the use of EdC, its transformation to EdUTP and EdUTP incorporation into DNA.

EdC is a deoxycytidine analogue that was originally used for the detection of replicated cells as an alternative to EdU^46^. Qu, et al. also showed that the use of EdC exhibits a less toxic effect on cells than the application of EdU commonly used for the detection of replicated cells^46^. In this respect, it was assumed that both EdC and EdU are incorporated into DNA. Later, it was shown that EdC is firstly transformed by cellular deaminases to EdU and as EdU incorporated into DNA. Previous data also strongly suggests that EdC itself is not, in contrast to EdU, effectively incorporated into DNA^29^. This finding represents a very important prerequisite for the detection strategy using the presented technique because the simultaneous incorporation of EdU and EdC would make it impossible to use click reaction as a detection system. Although antibody detection could be used instead because most of the anti-BrdU antibodies react with EdU^47^, click reaction is more cost-effective and does not require additional steps to make EdU accessible for the reaction with antibodies^43,47,48^.

It should be noted that a system based on other dCas can theoretically be used as well. A promising variant is, for example, 5-bromo-2′-deoxycytidine because the product of its deamination (5-bromo-2′-deoxyuridine) is effectively incorporated into DNA and can be detected in the DNA structure by specific antibodies^48,49^. Other such examples are 5-chloro-2′-deoxycytidine or 5-iodo-2′-deoxycytidine. The disadvantage of the mentioned system(s) is the necessity to use antibodies and procedures to reveal incorporated analogues in DNA structure^48,49^. It would also significantly increase the cost of such analyses.

The tested system provides the possibility to trap the product of deamination (EdU) in DNA, thereby enabling its direct visualisation in intact cells. This accelerates the whole procedure because no additional steps are necessary. In addition, EdU incorporated in DNA is almost insensitive to the unwanted reaction in cell lysates and therefore, even if cell lysis is required, it can be used without the risk of unwanted reactions. The data showed that both fluorescence microscopy and plate reader analysis provided similar results.

As the data indicated that EdC and EdCMP are effectively deaminated by CDD and dCMP deaminase, respectively, this technique can address both pathways. dCas are the targets of many metabolic pathways, therefore the extent of EdU incorporation reflects not only CDD and dCMP deaminase activities, but also the effectiveness of its transport by, for example, the human equilibrative nucleoside transporter 1 (hENT1)^50^, phosphorylation by rate-limiting deoxycytidine kinase (dCK), deoxycytidine monophosphate kinase, nucleoside diphosphate kinase^51^, and dephosphorylation of EdUMP by cytosolic 5′-nucleotidase II (cN-II)^51,52^. To suppress the effect of EdU metabolic branch and to better address the pathways on EdC site, the signal of incorporated EdU was calibrated with respect to EdU concentration. Although the impact of EdU pathways cannot be completely removed, the results showed that this enables comparison of the aggregated effect of EdC transport, dCK, CDD, and dCMP deaminase on EdC fate in different cell lines. Importantly, the presented technique allows very quick discrimination of those cell lines without CDD activity. In this respect, the developed method based on THU treatment provides the possibility to discriminate cells with and without CDD activity in less than 3 h after incubation with EdC or EdU. As the method does not require the simultaneous analysis of samples treated with serially diluted EdU, several cell lines can be analysed concurrently. This method is also relatively cheap and simple as it relies on the click reaction and conventional fluorescence microscopes or common plate readers for product detection.

In this respect, western blot analysis is much more time demanding as it is usually based on overnight incubation with antibodies. Moreover, cell lysates have to be prepared and the protein concentration determined, e.g.^53^. Western blot is also more expensive because antibodies, membranes, gels, and detection systems are required. Although RT-qPCR represents a relatively cheap and fast alternative, the cell lysis and RNA isolation, usually with commercial kits, is necessary^27^. It must be emphasised that a negative result for CDD activity based on the developed method does not generally imply a negative result for the western blot and/or RT-qPCR analyses because the missing activity can result from the presence of non-functional CDD.

The presented technique also showed that the overexpressed viral thymidine kinase substantially increases EdU signal in those cells lacking CDD due to its ability to convert deoxycytidine and its analogues to their monophosphate forms^45^. This confirmed the importance of this kinase for the efficient transformation of dCas to nucleoside monophosphate. As the 143B TK cell line provided very high EdU signal, even compared to those cell lines with high CDD amount, this emphasises the role of dCMP deaminase in the (in)activation route of dCas.

There is another interesting aspect to the possible application of the developed method for the measurement of EdU signal by a plate reader. The previously developed approach based on DAPI or Hoechst staining and signal enhancement by SDS enabled cytotoxicity evaluation in different cell lines^44^. In the presented study, this approach was adapted for the simultaneous analysis of EdU and DAPI signal to minimise the impact of the different numbers of cells among various samples. EdU signal was used for the estimation of the effectiveness of the pathways producing EdU from EdC and DAPI signal to correct the numbers of cells. As EdU is commonly used as a marker of replicating cells and the adapted approach based on the incubation of cells exclusively with EdU in the first step (Fig. 1b) provides both DAPI and EdU signal, it enables cytotoxicity testing simultaneously with the determination of DNA replication. From our data it is clear that the 10 μM EdU concentration usually provides sufficient signal to reveal replication activity in such an arrangement. The association of the two approaches into one procedure can therefore provide more information about the treated cells.

## Conclusion

A technique is described that enables the analysis of dCas fate based on EdC conversion followed by EdU incorporation into DNA. The data showed that this technique can be used to study CDD and dCMP deaminase pathways and also to analyse the role of deoxycytidine kinase in dCas metabolism. In this respect, a quick method, based on the use of EdC and CDD inhibitor, that enables the identification of cell lines missing CDD, is described.

### Supplementary Information

Supplementary Figures.

## Data Availability

All data supporting the results and conclusions are present in the manuscript. Additional data supporting the findings are available from the corresponding authors upon reasonable request.
