# Supplementary material for: A new technique for the analysis of metabolic pathways of cytidine analogues and cytidine deaminase activities in cells
Source: Sci Rep. 2023 Nov 22;13:20530. doi: 10.1038/s41598-023-47792-4 (PMC10665361; doi:10.1038/s41598-023-47792-4)

Supplementary Data

A new technique for the analysis of metabolic pathways of cytidine analogues and cytidine deaminase activities in cells

Anna Ligasová[1,*], Barbora Pisklákova[1,2], David Friedecký[1,2] and Karel Koberna[1,*]

[1] Institute of Molecular and Translational Medicine, Faculty of Medicine and Dentistry, Palacký University Olomouc, Olomouc, Czech Republic

[2] Laboratory of Inherited Metabolic Disorders, Department of Clinical Chemistry, Palacký University and University Hospital Olomouc, Olomouc, Czech Republic

***Corresponding authors**

Anna Ligasová - anna.ligasova@upol.cz

Karel Koberna - karel.koberna@upol.cz

Supplementary Figure 1 Original Western Blots for Figure 4
Figure 4a

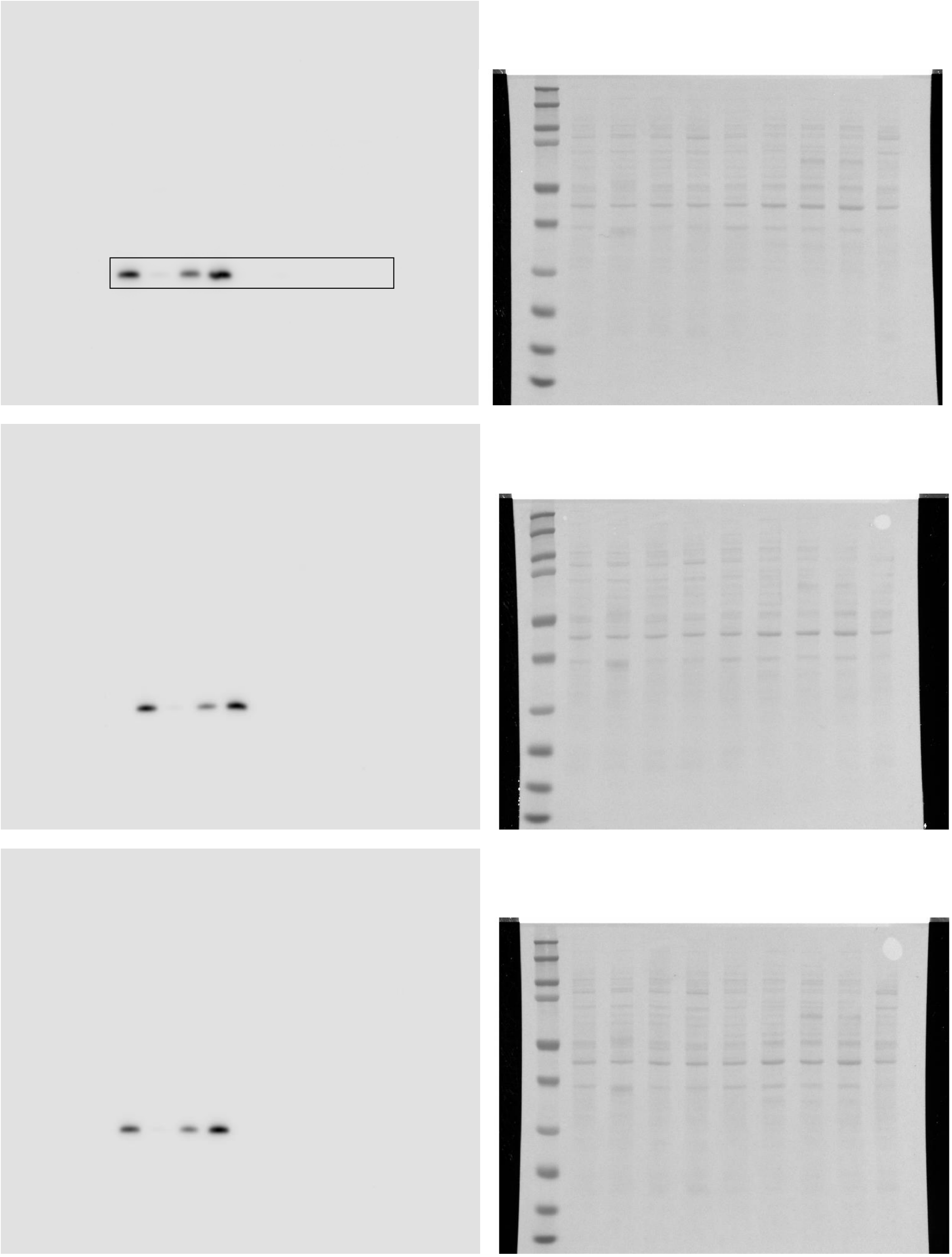

Figure 4b

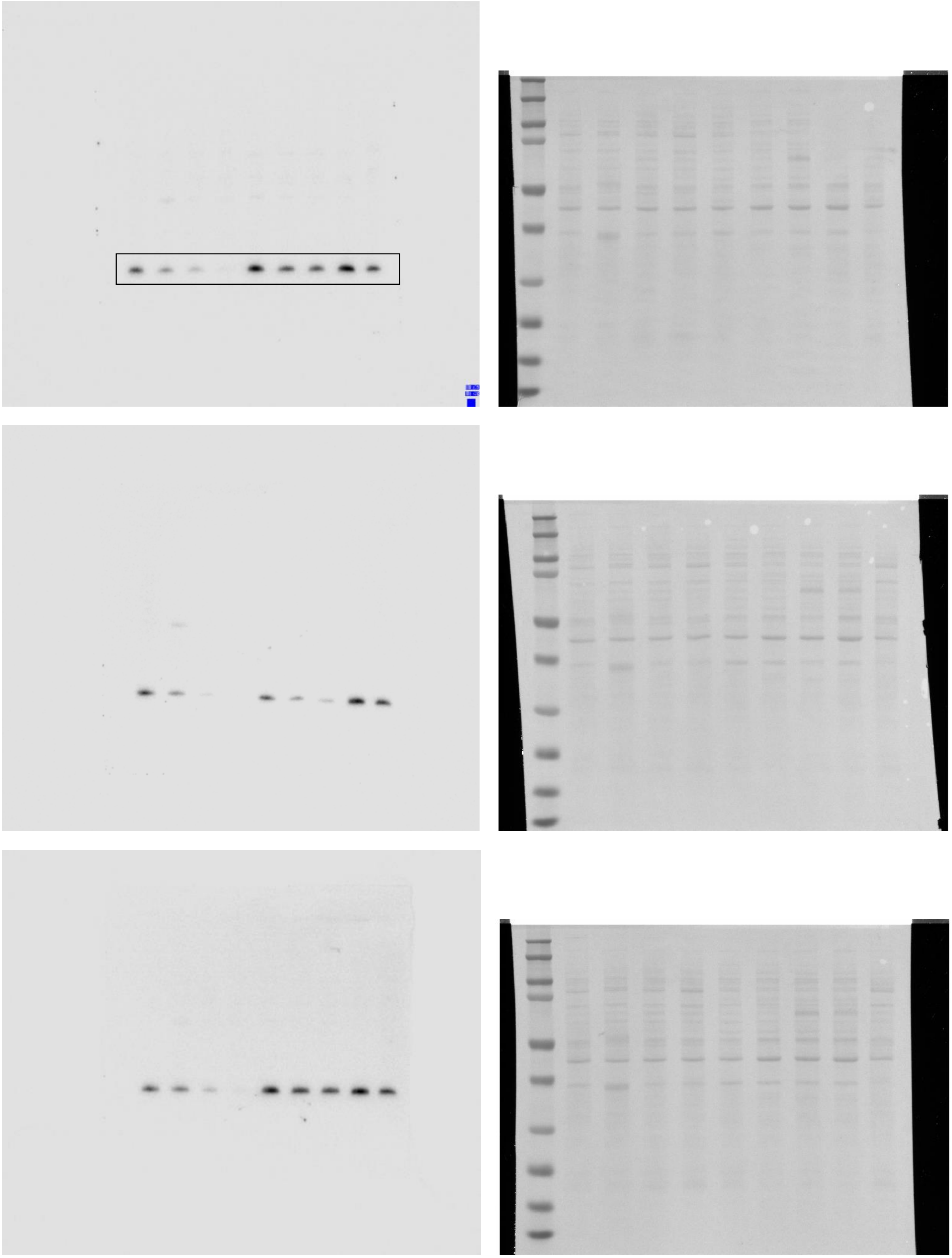

Supplement: Supplementary file 1 — Supplementary Figures. [file 41598_2023_47792_MOESM1_ESM.pdf]
